# Patient Portal Use Among Older Adults With Dementia Diagnosis

**DOI:** 10.1001/jamainternmed.2023.1568

**Published:** 2023-06-26

**Authors:** Kelly T. Gleason, Mingche M. J. Wu, Aleksandra Wec, Danielle S. Powell, Talan Zhang, Jennifer L. Wolff

**Affiliations:** 1 Johns Hopkins University School of Nursing, Baltimore, Maryland; 2 Johns Hopkins Bloomberg School of Public Health, Baltimore, Maryland

## Abstract

This cohort study assesses the level of engagement with an electronic health management system among patients with recently diagnosed dementia and their caregivers.

The patient portal has a growing role in health system navigation. Persons with dementia have especially complex health needs and less ability to perform electronic health management tasks than those without dementia.^1^ Persons with dementia and their care partners have a range of information needs that could be addressed through the patient portal,^2^ but little is known about patient portal practices in this population. We conducted a cohort study of older adults’ patient portal interactions at a large academic health system by receipt and timing of dementia diagnosis.

## Methods

We included patients aged 65 years or older with 2 or more evaluation and management visits during any 24 months within a 5-year period (October 3, 2017- October 2, 2022). The Johns Hopkins School of Medicine Institutional Review Board approved this study and waived the informed consent requirement because it used secondary data. We followed the STROBE reporting guideline.

Patient portal activity was computed from date- or time-stamped interactions, which contain login and session information.^3^ Portal user and used portal accounts were categorized as registered accounts with 1 or more logins. To measure portal activity, we created the portal activity metric, the ratio of number of portal sessions to number of clinical encounters. We examined the portal activity metric for all portal users and their proxy (with shared-access credentials) by dementia diagnosis. We then compared several measures of portal activity by older adults with recently diagnosed dementia each month for the 12 months before vs after diagnosis.

Statistical significance before vs after diagnosis was determined using a paired *t* test, with 2-sided *P* = .05 indicating significance. Statistical analysis was performed with Stata 17 (StataCorp LLC).

## Results

The cohort included 49 382 patients (mean [SD] age, 76.56 [8.60] years; 28 313 females [57.3%], 21 065 males [42.6%]) (Table). Only 6.4% of patients had diagnosed dementia. Those with a diagnosis were similarly likely to be registered for the patient portal (71.2% vs 71.5%; *P* = .69) but were more likely to have a registered care partner with shared access to their portal account (10.4% vs 3.3%; *P* < .001) than those without a diagnosis.

**Table.** Patient Portal Registration and Use by Dementia Status

Characteristic	Patients, No. (%)			*P* value^b^
	Total	Dementia diagnosis^a^		
		With	Without	
All patients	49 382 (100)	3170 (6.4)	46 212 (93.6)	NA
Patient characteristics				
Age, mean (SD), y	76.56 (8.60)	82.80 (8.27)	76.12 (8.43)	<.001
Female sex	28 313 (57.3)	2017 (63.6)	26 296 (56.9)	<.001^c^
Male sex	21 065 (42.6)	1153 (36.4)	19 912 (43.1)	
Race^d^				
Asian or Pacific Islander	1611 (3.3)	94 (3.0)	1517 (3.3)	<.001^c^
Black	11 427 (23.4)	957 (30.5)	10 470 (23.0)	
White	32 747 (67.2)	1947 (62.0)	30 800 (67.6)	
Other^e^	2953 (6.1)	141 (4.5)	2812 (6.2)	
Hispanic ethnicity^d^	723 (1.5)	40 (1.3)	683 (1.5)	<.001^c^
Married or partnered	27 053 (54.8)	1439 (45.4)	25 614 (55.4)	<.001^c^
Prefers English language	48 114 (97.4)	3055 (96.4)	45 059 (97.5)	<.001^c^
Patient portal registration				
Patient registered	35 311 (71.5)	2257 (71.2)	33 054 (71.5)	.69^c^
Care partner registered	1858 (3.8)	330 (10.4)	1528 (3.3)	<.001^c^
Patient portal use				
Patient portal user^f^	32 501 (65.8)	2075 (65.5)	30 426 (65.8)	.66
Portal activity metric, mean (SD)^g^	5.26 (6.37)	3.88 (5.10)	5.35 (6.44)	<.001
Mean (SD) No. of sessions^h^	150.52 (279.18)	131.17 (319.43)	151.85 (276.16)	<.001
Mean (SD) No. of messages sent from patient portal account^i^	29.18 (56.49)	28.77 (57.73)	29.14 (56.41)	.77
Mean (SD) No. of messages sent from shared access account^i^	15.09 (29.08)	19.50 (37.44)	13.85 (26.15)	.03
Mean (SD) monthly No. of patient portal sessions	5.61 (10.03)	5.77 (10.67)	5.60 (9.99)	.45
Mean (SD) monthly No. of patient portal messages	3.26 (3.77)	3.14 (3.22)	3.27 (3.80)	.14

Abbreviations: EMR, electronic medical record; NA, not applicable.

^a^ Dementia status was defined by Grodstein et al,^4^ with a diagnosis date based on the noted date recorded in the problem list.

^b^ Tests of statistical significance reflect the results from simple linear regression models or χ^2^ analyses comparing people with dementia diagnosis to people without dementia diagnosis.

^c^ Calculated using a χ^2^ test.

^d^ Race and ethnicity were obtained from the EMR of the health system.

^e^ Other included American Indian and unspecified. As a race option in the EMR, other was selected without further information.

^f^ A user was defined as the person who logged into the patient portal at least once over the 5-year study period.

^g^ Portal activity metric is operationalized by dividing the number of patient portal sessions (either by the patient or care partner with shared access credentials) by the number of clinical encounters within a 12-month period.

^h^ Metrics were computed for the 32 501 persons with an active patient portal over the 5-year study period.

^i^ Messaging was limited to medical advice request category, reflecting messages initiated by the patient or their registered proxy.

Persons with vs without dementia diagnosis had lower portal activity metric (3.88 vs 5.35; *P* < .001) but were similarly likely to be a portal user (65.5% vs 65.8%; *P* = .66) and sender of a similar number of messages from their portal account (28.77 vs 29.14; *P* = .77). More portal messages originated from registered care partners of patients with vs without a diagnosis (19.50 vs 13.85; *P* = .03) (Table).

Portal activity metric was significantly higher in the 12 months after than before dementia diagnosis (3.34 vs 2.02). Monthly number of messages sent (4.31 vs 3.32; *P* < .001) and number of sessions (6.54 vs 4.17; *P* < .001) were also higher in the 12 months after than before diagnosis (Figure).

**Figure.**
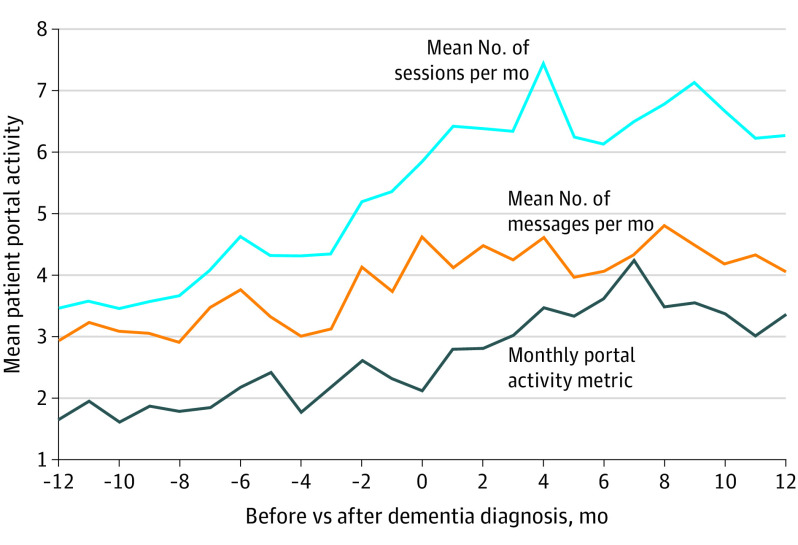
Measures of Patient Portal Activity in the 12 Months Before and After Dementia Diagnosis Portal activity metric is operationalized by dividing the number of patient portal sessions by the number of clinical encounters. A patient portal session is defined as logging in and conducting at least 1 other portal activity. Patient portal messages are the mean number of messages sent by active patient portal users in a given month.

## Discussion

This analysis found that older adults with and without diagnosed dementia were similarly likely to be registered for the patient portal. Although minimal in both groups, persons with dementia were 3 times more likely to have a registered care partner, and these care partners were actively engaged in messaging. Additionally, portal activity was significantly greater after a diagnosis.

A study limitation was use of electronic medical records, which are subject to missing and inaccurate data, including dementia, which is often underdiagnosed. We found that older adults with dementia and their care partners relied on the information and functionality afforded by the patient portal. These results, in conjunction with gaps in dementia care quality and the importance of care partner engagement and support,^5^ have implications for modalities of systems-level dementia care initiatives that leverage the patient portal, including efforts to remedy the low uptake of shared-access or proxy portal registration.^6^ Additionally, the results highlight the need to better support all patients, including those who desire or rely on care partners, through consumer-oriented health information technologies.

## Notes

Supplement.
Data Sharing Statement
