# Supplementary material for: Patient Portal Use Among Older Adults With Dementia Diagnosis
Source: JAMA Intern Med. 2023 Jun 26;183(8):881–3. doi: 10.1001/jamainternmed.2023.1568 (PMC10294010; doi:10.1001/jamainternmed.2023.1568)
Supplement: Supplement. — Data Sharing Statement [file jamainternmed-e231568-s001.pdf]

Data Sharing Statement

Gleason. Patient Portal Use Among Older Adults With Dementia Diagnosis. *JAMA Intern Med*. Published June 26, 2023. doi:10.1001/jamainternmed.2023.1568

Data
Data available: No

Additional Information
Explanation for why data not available: While aggregate data will be made available, the data used in this study would be identifiable if shared and can only be examined using a secure desktop by a IRB-approved study team member.
